# Validation of Activity Tracking Procedures in Elderly Patients after Operative Treatment of Proximal Femur Fractures

**DOI:** 10.1155/2018/3521271

**Published:** 2018-06-19

**Authors:** Hagen Schmal, Anders Holsgaard-Larsen, Kaywan Izadpanah, Jan Christian Brønd, Carsten Fladmose Madsen, Jens Lauritsen

**Affiliations:** ^1^Department of Orthopaedics and Traumatology, Odense University Hospital, Denmark; ^2^Department of Clinical Research, University of Southern Denmark, Denmark; ^3^OPEN, Odense Patient data Explorative Network, Odense University Hospital, Odense, Denmark; ^4^Department of Orthopedics and Trauma Surgery, Medical Center-Albert-Ludwigs-University of Freiburg, Faculty of Medicine, Albert-Ludwigs-University of Freiburg, Germany; ^5^Institute of Biomechanics and Sports Sciences, University of Southern Denmark, Denmark

## Abstract

**Background:**

Early postoperative physical activity in elderly patients suffering from proximal femoral fractures may reduce mortality. We hypothesized that activity trackers can reliably and objectively monitor the in-hospital mobilization, correlating with functional independence and quality of life.

**Methods:**

Three different tracker types (Fitbit™ flex, Misfit™ Shine, and Axivity AX3) at three locations (wrist, ankle, and femur) recorded steps and signal vector magnitudes (SVM) in 22 patients. They were 81 ± 8 years old, were equally distributed between the sexes, and had an ASA score of 2.5 ± 0.6. Single protocoled activity events (*n* = 191) were clinically categorized into 4 levels and correlated with the monitored signals. Additionally, 2 ± 1 and 8 ± 3 days after the operation, the EuroQol-5D and the Barthel-20 index supplemented this data.

**Results:**

All measurements at the wrist (Fitbit, Misfit) resulted in unacceptable accuracy; however, sensitivity and specificity reached around 90% using the Misfit at the ankle. Applying this combination, the correlation between real and measured steps (*R*^2^ = 0.99) and the category discrimination were statistically significant (*p* < 0.002). A discriminant analysis featured the calculation of four activity levels based on SVM measurements using the Axivity tracker at the femur. A cluster analysis showed a 100% agreement between the clinically observed and the calculated activity levels. The amount of active minutes or periods and both the EuroQol-5D and the Barthel-20 indices significantly increased between the analyzed time points after the operation. However, only the Barthel-20 was associated with the measured activity levels (*p* < 0.01).

**Conclusion:**

The Misfit and the Axivity trackers can reliably monitor activity in elderly patients after operative treatment of proximal femur fractures. However, the wear location is decisive. Objectively measured activity correlated with functional independence and quality of life.

## 1. Background

Proximal femoral fractures are common in elderly patients and are associated with a high mortality of around 25–30% within the first year [1]. This is determined by a variety of factors as comorbidity and age, which cannot or only partially be altered [2]. An influenceable key factor for a successful reintegration into normal life is the recovery of mobility. If patients succeed to regain their physical activity, they have a higher chance to survive [3]. Unfortunately, an increased age is often accompanied by a decrease of mental capabilities, which makes it difficult to correctly assess health and physical status of these elderly patients. Therefore, typical questionnaires as the Oswestry Disability Index [4] or the EuroQol 5D [5] (EQ-5D, https://euroqol.org/) have limited capacities to evaluate this population. Activity trackers that are also known as step counters or wearables have recently obtained growing interest for surveillance of motion and physical activity and have been successfully validated in young people [6–8]; however, differences between various manufacturers were found [9]. Until now, a validation for older people and for postoperative monitoring is lacking. Since clinical trials in the elderly and operated patients are in need of objective evaluation parameters, which can easily be obtained and are not dependent on active cooperation, activity trackers appear to be ideal tools for this frail population. We hypothesize that wearables are able to monitor activity in elderly patients and supply personalized information about their physical motions. The study aimed to validate the feasibility of activity trackers for this frail patient group and to adjust the algorithms of analysis and the normal values for the different activity categories (bedrest during sleep/night, bedrest awake/during the day, sitting and mobilization/transfers, and physiotherapy). Another aim was to analyze whether the measured activity parameters correlate with the results of classical instruments evaluating functional independence (Barthel 20) and quality of life (EQ5D).

## 2. Methods

### 2.1. Evaluated Trackers

Two basically different tracker types were tested. First, commercially available step counters were chosen based on recent evaluations [9], namely, the Fitbit flex (San Francisco, Ca, USA) and the Misfit Shine (Burlingame, CA, USA). These devices measure only the absolute number of steps, and the algorithm, i.e., how accelerometer signals are converted into calculated step counts, is unknown. The trackers were connected via Bluetooth to a mobile smartphone app, which allowed the read-out of steps performed. Secondly, a 3-axis accelerometer, Axivity AX3 (Newcastle upon Tyne, UK) was evaluated, which had the possibility of analyzing the raw accelerometer data and to develop and apply an own, adapted algorithm for activity measurement based on the calculated signal vector magnitudes (SVM) using the following equation: $\text{abs}(\sqrt{\left(x^{2}+y^{2}+z^{2}\right)}-1$).

The placement of the devices for evaluation was as follows: the Fitbit flex and the Misfit Shine on the wrist and on the ankle, using the commercially available bracelets; and the Axivity AX3 was skin-taped at the lateral distal femur as recently described [10]. The actual steps made were counted during the observation and video recorded. Data from the Axivity AX3 were recorded in 60-second epochs applying a filter between 0.5 and 20 Hz and subjected to a wear time analysis after continuous data registration. The tracker was subsequently connected to a personal computer using an USB-interface. The analysis was done by the standard software ([AX3] OMGUI Configuration and Analysis Tool, https://github.com/digitalinteraction/openmovement/wiki/AX3-GUI).

### 2.2. Stages of Evaluation

The analysis was done in two stages. First, a validation in a postoperative in-hospital setting was carried out based on a comparison with golden standards such as the actual steps made and signaling during specified periods with clinically defined activity levels. Hereby, SVM measured with the Axivity AX3 were categorized following a threshold adaption. While this technique allowed measurement of physical activity in the different categories and the number of active minutes per day, Misfit Shine and Fitbit flex allowed only registration of steps.

Secondly, the reliability during use in the daily routine work was compared with current standardized scores, namely, the Barthel-20 index [11] and the EQ5D-3L [12].

### 2.3. Measurements

#### 2.3.1. Definition of Categories for Physical Activity and Verification for Step Count Measurement

Recent studies reported important differences between various methods of activity measurement, which were dependent on specific occupations [13]. Therefore, activity categories were defined as follows: bedrest during sleep/night (“no” or category 1 activity), bedrest awake/during the day (“low” or category 2 activity), sitting in chair and mobilization outside the bed including transfers (“middle” or category 3 activity), and physiotherapy including shuffling gait (“high” or category 4 activity). A bedside protocol facilitated registration of all events, which were recorded by the nursing staff and physiotherapists. Additionally, all category 3 and 4 events were observed by the facilitator of the study, counting the actual steps during mobilization. The conditions for the preceding power analysis were defined as follows: assuming a 25% difference between the different levels of activity, a standard deviation of 20%, and a 2-sided confidence interval of 95%, the inclusion of 8 patients with one event in each category (a total of 32 events) would have provided the necessary power of 80%. If at least one step was registered during a defined event, this period was recognized as an active event. This was the basis for the calculation of sensitivity and specificity for the different step counters and wear locations. The higher the activity level was (or the more intense patients were moving), the less was the time patients could keep up with the activity level. The average times for event durations reflect this circumstance, which were 175 ± 123 minutes for category 1; 89 ± 141 minutes for category 2; 59 ± 49 minutes for category 3; and 16 ± 8 minutes for category 4. Shortly summarized, the older patients got quickly tired.

#### 2.3.2. Continuous Measurement during the Hospital Stay

For monitoring of activity during the hospital stay, the patients were equipped with a Misfit Shine at the ankle and an Axivity AX3 at the lateral femur of the unaffected side. At day 2 ± 1 (shortly after the operation) and 8 ± 3 (shortly before discharge) activity signals were analyzed by calculating the mean of all acquired 24-hour-data during these periods. Correlating with these two data sets, the EuroQol 5D-3L questionnaire and the Barthel-20 handicap index were monitored. Epidemiological characteristics such as age, sex, body mass index (BMI), the degree of mobility (bedridden, need for support or normal), ASA classification (physical status according to the American Society of Anesthesiologists), and treatment characteristics such as the type of fracture, the type of operation, the time between the fracture occurred and operation supplemented the data for activity, functional status, and quality of life were also registered for analysis.

### 2.4. Participants

#### 2.4.1. Inclusion Criteria

- Existence of a proximal femoral fracture (location AO 31) and successful operation
- Age ≥ 65 years
- Being able to read and understand Danish
- Informed consent

#### 2.4.2. Exclusion Criteria

- Open fractures
- Polytrauma
- Colonization with multiresistant bacteria
- Preoperatively bedridden patients
- Infection of the wound

### 2.5. Characteristics and Operative Treatment

Twenty-two patients (*n* = 22) undergoing surgery because of proximal femur fracture and an average age of 81 ± 8 years were analyzed. 50% were male or female; the average ASA score was 2.5 ± 0.6. 59% of the patients, suffering from an intertrochanteric fracture, were treated by an osteosynthesis using a Gamma3® nail (*n*_GN_ = 8, Stryker, Kalamazoo, MI, USA) or a Dynamic hip screw (*n*_DHS_ = 3, DePuy Synthes Companies, Zuchwil, Switzerland). In displaced femoral neck fractures, a bipolar hemiarthroplasty or a total hip (*n*_HA_ = 9, CORAIL®, DePuy Synthes Trauma, West Chester, PA, USA) was implanted. Nondisplaced femoral neck fractures were stabilized using 8 mm cannulated Olmed screws (*n*_OS_ = 2, Biomet, Warsaw, IN, USA) as previously described [14]. All operations allowed full weight bearing. Indications and operations followed the local standard operating procedures. The average BMI was 25.9 ± 5.4; 64% of the participants were in need of mobility aids before their fall.

### 2.6. Registration

The project was registered and approved by the Ethical Board Region Southern Denmark (Project-ID S-20150193). Furthermore, the project was approved according to the Act on Processing of Personal Data (Journal no. 15/53376). The use of the EQ5D questionnaire was registered at the EuroQol Research Foundation's website (ID 24787).

### 2.7. Data Management and Statistics

REDCap™ (Research Electronic Data Capture), a secure application for online surveys and databases, facilitated data management, which is supported by the OPEN initiative (Odense Patient data Explorative Network). The project got licensed by OPEN (Journal no. 15/52741). First, an analysis of variance (ANOVA) was applied to make multiple comparisons between numerical datasets of different categories. Then normally distributed numeric data was compared using the paired (Student's) *t*-test. Otherwise or in case of nonnumeric data, the Mann–Whitney *U* test determined the significance of difference. For correlations, the Spearman *ρ* or the Pearson *R* was calculated depending on the kind of variable. If appropriate, a regression analysis was additionally calculated. Category definitions were facilitated by a discriminant and a cluster analysis as recently shown [15]. A cluster analysis determines the belonging of a value to a group based on the distance between the values. Unlike in cluster analysis, the discriminant analysis was used after the groups have already been defined, calculating the spread and the reliability of group membership for the values of physical activity compared to the clinical classification. The sensitivity was calculated as the “true positive rate” and the specificity as the “true negative rate”. Incidences are compared using the chi square test. The significance level was set as usual at 0.05.

## 3. Results

### 3.1. Commercial Wearables and Step Count Analysis

**Figure 1:**
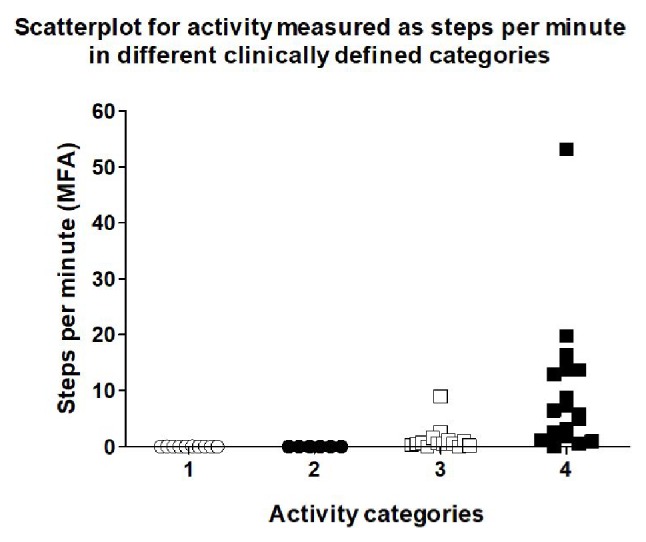
The chart shows the steps per minute during different clinically defined activity categories using a Misfit Shine worn at the ankle (MFA).

**Figure 2:**
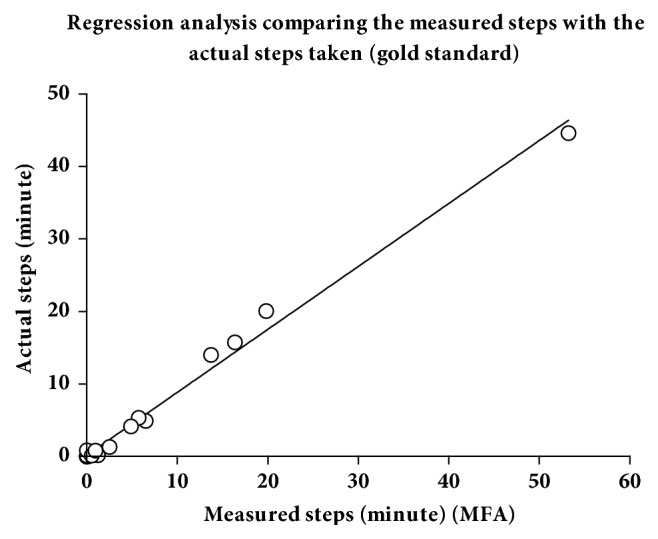
The chart shows a regression analysis correlating the steps per minute measured with the steps per minute actually made (gold standard). The steps were recorded by a Misfit Shine worn at the ankle (MFA). *R*^2^ = 0.99, *n* = 24.

During 121 protocoled activity events, physical activity was analyzed by registered steps. Furthermore, the actual steps made by patients during physiotherapy were counted. Sensitivity and specificity for the Misfit Shine and the Fitbit flex at two different locations (wrist and ankle) were calculated and compared (Table 1). Regarding correct activity event detection, all measurements at the wrist resulted in unacceptable low sensitivity or specificity. Both parameters ranged at 60% and 57%, respectively, when analyzing the results measured by the Fitbit flex device at the ankle. Although this was better than the measurements at the wrist, the values still were unacceptable. Therefore, the analysis of Fitbit flex was stopped at this stage of evaluation. Sensitivity reached 91% and specificity 88% for the Misfit Shine at the ankle.  Figure 1 depicts the steps per minute registered during events with different activity levels. Although some signals were also measured during category 3 events, only the values registered in category 4 using the Misfit tracker at the ankle could be statistically significantly discriminated from the other activity levels (ANOVA *p* < 0.05, category 3 versus 4 *p* = 0.0012).  Figure 2 depicts a regression analysis of the steps really made with the steps registered using he Misfit Shine at the ankle (*n* = 24). The correlation was statistically highly significant (*R*^2^ = 0.99, *p* < 0.0001).

### 3.2. Axivity AX3 Accelerometer Analysis

**Figure 3:**
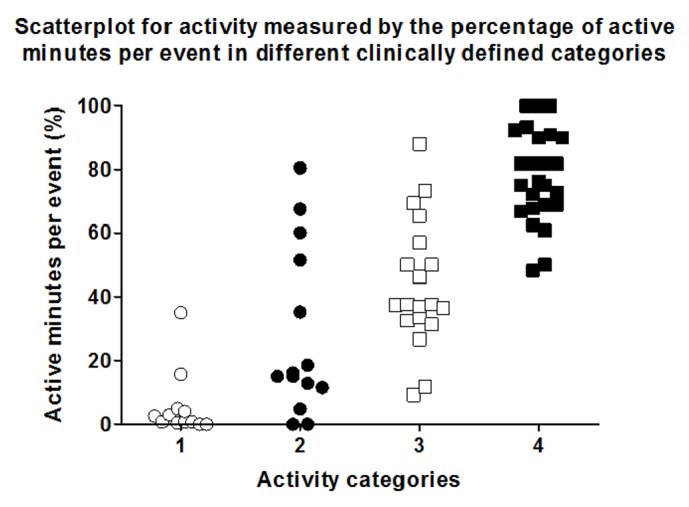
Scatterplot showing measured activities categorized according to clinical activity definitions. The graph represents data, which show percentages of active minutes per event. The decision, whether a minute was active or not, was based on a SVM cut-off calculation.

**Figure 4:**
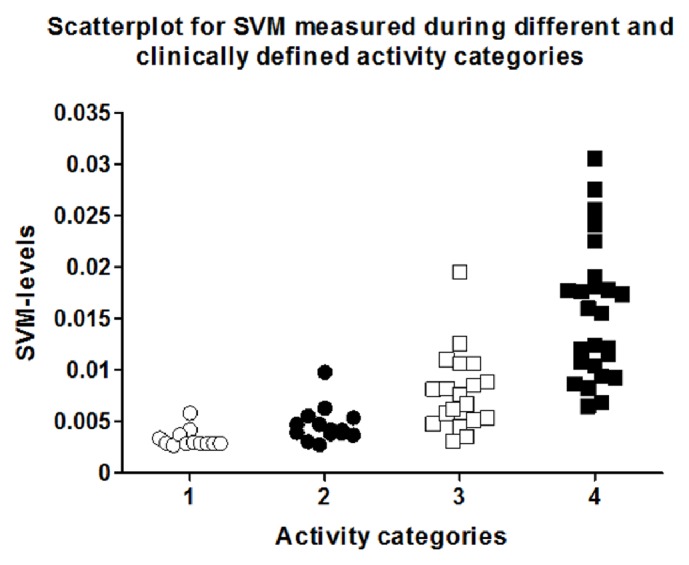
Scatterplot showing measured SVM categorized according to clinical activity definitions.

**Figure 5:**
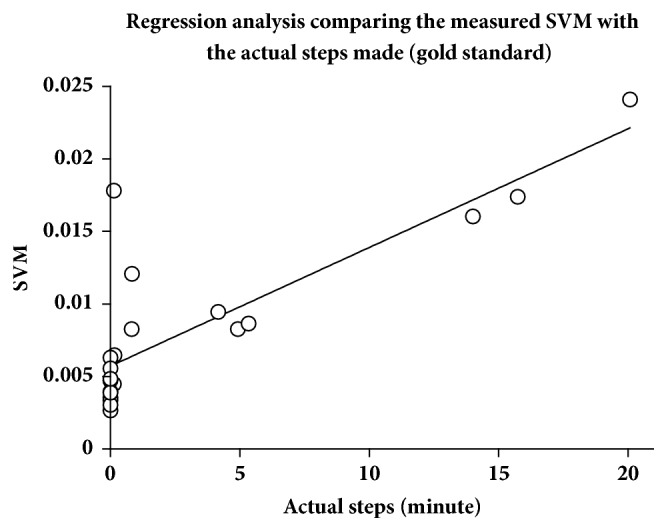
The chart depicts a regression analysis correlating the measured Signal Vector Magnitude (SVM) with the steps per minute actually made (gold standard). The steps were recorded by Axivity tracker worn at the lateral femur. *R*^2^ = 0.68, *n* = 22.

The calculation of SVM facilitated the analysis of Axivity AX3 accelerometer signals (*n* = 70). Hereby, the 90th percentile of 12 category 1 events defined the threshold between being active or not active in the examined slow walking population (SVM = 0.005). Afterwards, every minute of each event was categorized as an active or not active minute. The relative frequency of active minutes during one event was grouped with the clinically defined activity level. These data pairs underwent a discriminant analysis to determine category intervals, resulting in the following definitions: category 1 (“no activity”) 0–10%, category 2 (“low activity”) > 10–25%, category 3 (“middle activity”) > 25–60%, and category 4 (“high activity”) > 60%. This resulted in a correct grouping of 74.3% of the measured accelerometer signals, when the clinical definition was set as the gold standard.  Figure 3 shows the distribution of the calculated percentage of active minutes per event in the different categories. The cluster analysis showed a 100% agreement between the clinically observed activity and the monitored, clustered SVM-levels. This was caused by the fact that the data were arranged patient-wise, and patients ranging at the lower boarder on a category did the same in all other categories. The average values for the relative frequency of active minutes reached 5.63 ± 10.2% for category 1, 27.76 ± 26.5% for category 2, 43.68 ± 20.2% for category 3, and 77.94 ± 14.6% for category 4. The statistical variance analysis could significantly discriminate between all categories (Table 2, *p* < 0.05). The same analysis was done using the plain average SVM-levels per event without using the threshold analysis. This resulted in the following definitions: category 1 (“no activity”) 0–0.0047, category 2 (“low activity”) > 0.0047–0.0072, category 3 (“middle activity”) > 0.0072–0.01, and category 4 (“high activity”) > 0.01. By this, only 57.1% could be classified correctly, and the cluster analysis resulted in only 83% agreement.  Figure 4 depicts the scatterplot for the SVM values measured during the 4 different activity categories. The ANOVA analysis for this distribution discriminated only 3 categories, because there was no statistically significant difference between categories 1 and 2 (Table 3). Since SVM-signals are typically converted into steps, a regression analysis for registered SVM and real steps was performed, resulting in a *R*^2^ of 0.68.  Figure 5 shows the correlating graph. Applying a threshold of 1 step/minute, the *R*^2^ improved to 0.98.

### 3.3. Continuous Monitoring of the Hospital Stay

**Figure 6:**
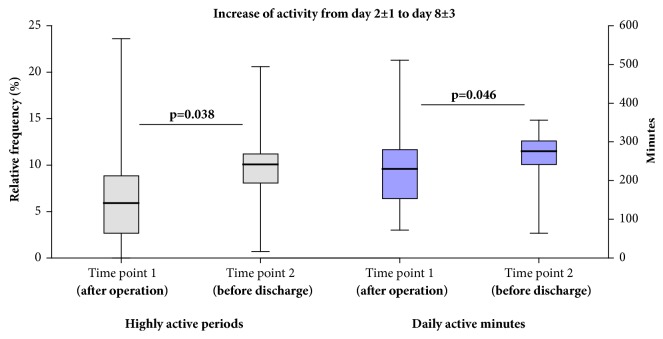
After an operation for stabilization of the proximal femur, the activity is increasing during the first couple of days. This can be measured using an Axivity tracker and calculating the highly active periods (category 4) per day (%, left) or the active minutes per day (right).

**Figure 7:**
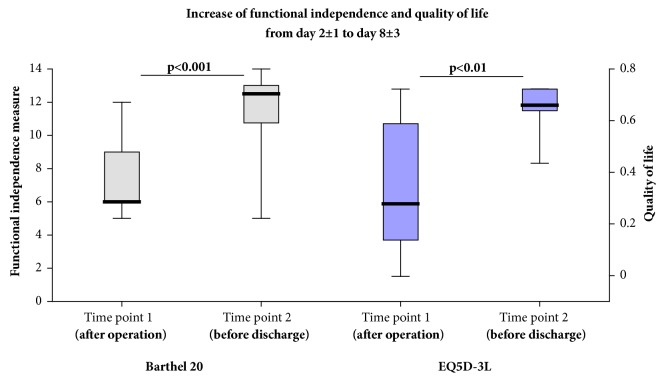
After an operation for stabilization of the proximal femur, the functional independence measured by the Barthel 20 index (left) and the quality of life measured by the EQ5D-3L (right) is increasing during the first couple of days.

During the next validation stage, the applicability of activity measurement was tested over the period of a hospital stay, comparing two different time points, day 2 ± 1 (shortly after the operation) and 8 ± 3 (shortly before discharge). The established scores for quality of life EQ5D-3L and functional independence Barthel 20 served as comparisons. While the activity in the categories 1–3 did not show statistically significant differences, the portion of highly active periods (category 4, 7.4 ± 6.6% versus 9.8 ± 5.2%) and the total number of active minutes (234 ± 121 minutes versus 256 ± 80 minutes) increased significantly (Figure 6). Similarly, the number of active minutes in the category 4 according to SVM grouping increased from 63 ± 33 min to 99 ± 54 min (*p* = 0.022). Similarly, the Barthel 20 index increased from 7.5 ± 2.4 to 11.5 ± 2.7 (*p* = 0.003) and the EQ5D-3L from 0.36 ± 0.28 to 0.65 ± 0.08 (*p* = 0.004) (Figure 7). However, only the Barthel 20 index at time point 1 was associated with the primarily measured activity levels (*R*^2^ = 0.52, *p* = 0.006). Otherwise, there was no statistically significant association between activity parameters and functional independence or quality of life.

## 4. Discussion

The most important finding of this study is the validation of methods to measure activity in patients with proximal femur fractures using the Misfit Shine and the Axivity AX3 trackers. However, algorithms discriminating 4 activity categories needed to be adapted to the functional level of this slow-moving population, and reliability does not only depend on the hardware but also strongly on wear locations. A regression analysis indicated that objectively measured physical activity is a separate construct of outcome besides patient reported quality of life or need for care to evaluate individual recovery progress.

Several positive effects are attributed to physical activity for the present patient group; if practiced on a moderate level, among others, the risk for hip fractures is reduced [16, 17]. Furthermore, activity has a positive influence on the bone mineral density in 70-year-old people in bones, which are mechanically stressed during walking [18]. This becomes especially important in elderly people, suffering from proximal femur fractures with a high risk for a second fall and a high mortality. However, the physical activity of these patients is very low, which makes it impossible to transfer data from a normally moving population [19]. As a matter of fact, our data show a 300x decreased activity compared to young people such as women of childbearing age [19]. This is based on the categorizing SVM values and associated with a technical challenge, because the developed algorithms and thresholds need an adaption, if meaningful results describing differences between patients shall be retrieved from measurements in this population. Likewise, a recent publication described a failure rate in a pedometer analysis in a slow walking population going 1 km/h [18], which is close to the patients walk in a postoperative phase following proximal femur fractures. Despite this, an objective tool to monitor activity would be important, because it signals progress and individual rehabilitation needs and does not require cognitive cooperation of the patient. Therefore, these devices also add a potential safety feature to the treatment by controlling the individual course of healing regarding physical activity, but of course only, if the tracker measures the present low activity levels with a high validity.

While in younger patients with a high activity level the wear location does not seem to play a decisive role for the registered accelerometer signals [8], we found a highly significant influence in older individuals with proximal femur fractures. This becomes clear by observing their movement patterns, because they are getting mobilized using a walking frame, which they hold and utilize to relieve weight bearing on the operated leg. The influence of wear location was recently demonstrated also in patients undergoing hip arthroplasty [20]. Principally, they are allowed to have full weight bearing, but, due to pain, this is frequently difficult to realize, especially during the first couple of days after surgery. Moreover, the steps made are small and may be categorized as shuffling gait that usually are not associated with an accompanying acceleration of the body center of mass above a cut-off threshold defining a step. Contrariwise, caused by the slow walking velocity and the support by a walking frame, elderlies rather avoid moving the hip and the upper body. Nevertheless, plain step measurement using the Misfit Shine at the ankle provides very valid results regarding the actual number of steps made. However, this only allows discriminating between two activity categories, walking and not walking. This does not necessarily sufficiently monitor postoperative recovery in this slow (or non) walking population with high responsiveness, because all other activities fall under the cut-off and are not registered as activity. In contrast, the cut-off analysis, which was based on plain Axivity tracker SVM measurements, allowed not only to discriminate 4 different levels of clinically defined activity, but also statistically significantly demonstrated postoperative recovery similar to classical instruments as the Barthel 20 or the EQ-5D indices. The analysis based on periods was thought to better correlate with registered activities such as a short walk followed by relaxing in a chair. However, not only the number of highly active periods but also the number of highly active minutes could show the expected progress in activity during the postoperative period. This indicates that the length of the analyzed period is less decisive than the definition of the threshold for activity. In fact, EQ-5D demonstrated a good reliability to describe the influence of secondary shortening of the femoral neck on quality of life [12]. In our study, this index also significantly increased during the short-term postoperative rehabilitation phase. However, there was no statistically significant correlation between the parameters characterizing postoperative activity as the amount of active minutes or steps made with the EQ-5D. Similarly, the Barthel 20 index is a valid predictor for outcome after proximal femur fractures regarding mortality, residential status, and independent walking ability [11]. Although we found a significant correlation between our activity parameters and the Barthel 20 index shortly after the operation, this could not be confirmed for the later time point before discharge. Despite lacking statistical association, both activity and Barthel 20 increased significantly. We therefore conclude that activity is an independent factor besides functionality (Barthel 20) and quality of life (EQ-5D) for evaluation of recovery after proximal femur fractures. In contrast to the EQ-5D index, activity can be measured automatically and objectively and does not require active participation of the patient. Though the Barthel 20 index can be evaluated by the nursing staff and does not depend on patients' cooperation, it also cannot be generated automatically. In future, activity trackers may therefore offer the possibility for an objective evaluation of different operative treatment options that aim to increase mobility. It also supports personalized treatment concepts and a distribution of resources according to patients' requirements. However, as shown by the results of this study, activity measurement is connected to certain prerequisites such as a validation for the needs of the specific target group.

## 5. Conclusion

In a population of elderly patients with proximal femur fractures the use of Axivity trackers offers a variety of advantages compared to simple step counts when measuring postoperative activity. It allows adapting SVM thresholds, grouping physical activity in different categories, retracing read-outs, and wear time verification. Hereby, steps may be calculated, but mobilization in a chair can also be registered. Furthermore, additional parameters as temperature are available and thresholds can be optimized for any other population, ensuring maximal flexibility.

## Figures and Tables

**Table 1:** Comparison of sensitivity and specificity of two different tracker devices (Misfit Shine and Fitbit flex) at two different locations (wrist and ankle). The fifth column (“Correlation Real *R*square”) indicates the result of a regression analysis correlating the registered steps with real steps made (gold standard). The sixth column (“ANOVA Discrimination”) reports the results of an ANOVA regarding the discrimination of different activity categories by means of the indicated tracker configuration. MFA: Misfit Shine worn at the ankle, FBA: Fitbit flex worn at the ankle, MFW: Misfit Shine worn at the wrist, and FBW: Fitbit flex worn at the wrist.

Method	*n*	Event detection Sensitivity	Event detection Specificity	Correlation Real *R*square	ANOVA Discrimination
MFA	48	91	88	0.99	category 4
FBA	25	60	57	n.s.	n.s.
MFW	11	100	0	n.s.	n.s.
FBW	37	16	83	0.98	n.s.

Sum	121				

**Table 2:** ANOVA for percentages of active minutes per event distributed according to clinically defined activity categories. The decision, whether a minute was active or not, was based on a SVM cut-off calculation. The table shows the critical average differences between group pairs (right up) and the statistical significance evaluation (*p* < 0.05) left down.

Activity category		1	2	3	4
	Average for % of active minutes				
1	5.63	-* *-* *-* *-	14.57	13.66	13.01
2	27.76	yes	-* *-* *-* *-	13.05	12.37
3	43.68	yes	yes	-* *-* *-* *-	11.28
4	77.94	yes	yes	yes	-* *-* *-* *-

**Table 3:** ANOVA for SVM distributed according to clinically defined activity categories. The table shows the critical average differences between group pairs (right up) and the statistical significance evaluation (*p* < 0.05) left down.

Activity category		1	2	3	4
	Average for SVM				
1	0.0034	-* *-* *-* *-	0.0036	0.0034	0.0032
2	0.0047	no	-* *-* *-* *-	0.0032	0.0031
3	0.0080	yes	yes	-* *-* *-* *-	0.0028
4	0.0155	yes	yes	yes	-* *-* *-* *-

## Data Availability

The datasets supporting the conclusions of this article are included within the article and its supplementary materials (Category_calculation.csv and Activity_data_time_points.csv).

## Abbreviations

ANOVA: Analysis of variance
AO (Foundation): Arbeitsgemeinschaft für Osteosynthesefragen
ASA: American Society of Anesthesiologists
EQ: Euroqol (https://euroqol.org/)
FBA: Fitbit flex worn at the ankle (FitBit Ankle)
FBW: Fitbit flex worn at the hand wrist (FitBit Wrist)
MFA: Misfit Shine worn at the ankle (MisFit Ankle)
MFW: Misfit Shine worn at the hand wrist (MisFit Wrist)
REDCap™: Research Electronic Data Capture.

## Supplementary Materials

Supplementary Materials Category_calculation.csv and Activity_data_time_points.csv is file containing data sets.
